# Evaluation of a clinical method for selective electrode deactivation in cochlear implant programming

**DOI:** 10.3389/fnhum.2023.1157673

**Published:** 2023-03-30

**Authors:** Sarah E. Warren, Samuel R. Atcherson

**Affiliations:** ^1^Cochlear Implant Research Laboratory, School of Communication Sciences and Disorders, University of Memphis, Memphis, TN, United States; ^2^Department of Audiology, Arkansas Children’s Hospital, Little Rock, AR, United States; ^3^Department of Audiology and Speech Pathology, University of Arkansas for Medical Sciences, Little Rock, AR, United States; ^4^Department of Otolaryngology–Head and Neck Surgery, University of Arkansas for Medical Sciences, Little Rock, AR, United States

**Keywords:** cochlear implant, programming, electrode deactivation, speech perception, pitch ranking, clinical practice

## Abstract

**Background:**

Cochlear implants are a neural prosthesis used to restore the perception of hearing in individuals with severe-to-profound hearing loss by stimulating the auditory nerve with electrical current through a surgically implanted electrode array. The integrity of the interface between the implanted electrode array and the auditory nerve contributes to the variability in outcomes experienced by cochlear implant users. Strategies to identify and eliminate poorly encoding electrodes have been found to be effective in improving outcomes with the device, but application is limited in a clinical setting.

**Objective:**

The purpose of this study was to evaluate a clinical method used to identify and selectively deactivate cochlear implants (CI) electrodes related to poor electrode-neural interface.

**Methods:**

Thirteen adult CI users participated in a pitch ranking task to identify indiscriminate electrode pairs. Electrodes associated with indiscriminate pairs were selectively deactivated, creating an individualized experimental program. Speech perception was evaluated in the baseline condition and with the experimental program before and after an acclimation period. Participant preference responses were recorded at each visit.

**Results:**

Statistically significant improvements using the experimental program were found in at least one measure of speech perception at the individual level in four out of 13 participants when tested before acclimation. Following an acclimation period, ten out of 13 participants demonstrated statistically significant improvements in at least one measure of speech perception. Statistically significant improvements were found with the experimental program at the group level for both monosyllabic words (*p* = 0.006) and sentences in noise (*p* = 0.020). Additionally, ten participants preferred the experimental program prior to the acclimation period and eleven preferred the experimental program following the acclimation period.

**Conclusion:**

Results from this study suggest that electrode deactivation may yield improvement in speech perception following an acclimation period. A majority of CI users in our study reported a preference for the experimental program. This method proved to be a suitable clinical strategy for identifying and deactivating poorly encoding electrodes in adult CI users.

## 1. Introduction

Cochlear implants (CI) are surgically implanted neuroprosthetic devices which allow for open-set speech perception in individuals who obtain limited benefit from conventional amplification. While CIs provide an improvement in auditory ability to virtually all individuals who qualify and properly use the device, speech perception outcomes remain highly variable among this population (Sarant et al., 2001; Boisvert et al., 2020; Heutink et al., 2021).

Cochlear implants systems work by filtering incoming auditory signals into frequency bands and delivering electrical pulses through contacts placed along the cochlea in a tonotopic organization mimicking a healthy cochlea. Ideally, each contact will stimulate a distinct neural population that will yield independent channels of stimulation with a high degree of spectral independence from adjacent electrodes, thus resulting in distinctive pitch percepts along the basilar membrane. In reality, a high degree of individual variability is related to a suboptimal electrode-neural interface. The primary peripheral factors that contribute to variability in the electrode-neural interface include spiral ganglion survival patterns (e.g., Pfingst et al., 2011) and intracochlear electrode placement (e.g., Finley et al., 2008). A poor electrode-neural interface can result in an overlap in stimulation known as channel interaction. Some degree of channel interaction is expected as the cochlea is filled with highly conductive fluid; however, excessive overlap of stimulation due to suboptimally placed electrodes results in perceptually indiscriminate channels of stimulation (e.g., Finley et al., 2008; Bierer, 2010; Ramos de Miguel et al., 2018). Indiscriminate channels result in reduced spectral resolution abilities and, consequently, poor speech perception performance. Advancements in radiologic imaging over the past two decades have allowed researchers to describe the relationship between electrode placement and speech perception ability (e.g., Skinner et al., 2007; Finley et al., 2008; Holden et al., 2013). The theory of channel independence is well documented, as a number of studies have found that (1) suboptimal intracochlear electrode location is associated with poorer speech perception ability (Finley et al., 2008; Holden et al., 2013), and (2) electrode discrimination ability is correlated with speech perception ability (Dawson et al., 2000; Kenway et al., 2015; Mathew et al., 2017, 2018; Biesheuvel et al., 2019).

A range of behavioral and objective techniques have been developed to identify poorly encoding electrodes. The most common behavioral measures include electrode discrimination (e.g., Zwolan et al., 1997), pitch scaling (e.g., Saleh et al., 2013; Vickers et al., 2016), and modulation detection (Garadat et al., 2013). Objective methods of identifying poorly encoding electrodes include the auditory change complex as an objective measure (e.g., Mathew et al., 2018) and CT-imaging to evaluate scalar location (e.g., Noble et al., 2014). Once a poorly encoding electrode has been identified, an electrode can be selectively deactivated, allowing the frequency allocation table (FAT) to adjust so that electrical current is delivered to areas of robust neural populations, resulting in discrete neural stimulation. Several studies have investigated the effects of CI programs following the deactivation of poorly encoding electrodes, and results have been mostly positive with some instances of mixed or poor outcomes. Findings from several studies suggest that deactivating poorly encoding electrodes is associated with user improvement in some measures of speech perception (Zwolan et al., 1997; Saleh et al., 2013; Noble et al., 2014; Danieli et al., 2021), spectral resolution (Noble et al., 2014; Labadie et al., 2016; Zhou, 2017), and subjective sound quality (Noble et al., 2014; Danieli et al., 2021). Conversely, other studies reported no improvements in outcomes for some participants following electrode deactivation (Henshall and McKay, 2001; Vickers et al., 2016; Debruyne et al., 2017), and in some conditions, poorer outcomes in speech perception (Vickers et al., 2016), spectral ripple discrimination (Debruyne et al., 2017), and patient preference (Debruyne et al., 2017). These differences in outcomes can be attributed to a range in methodologies for deactivation criteria as differences in participant characteristics such as electrode scalar location, device design, and a person’s auditory history. For example, it is noted in Vickers et al. (2016) that the lack of improvement experienced by CN users is likely related to the use of n-of-m strategies, as not all channels are stimulated in each cycle, therefore reducing the sensitivity to channel overlap. Based on this evidence, the benefits of electrode deactivation are promising but likely highly individualized. These methods should be applied cautiously and with specific patient characteristics and preferences in mind. Additionally, the methodologies of these studies involve equipment that may require technical training or initial investment, establishing another clinical barrier to implementation.

While evidence of the benefits of electrode deactivation in some CI users have been established for decades, this practice is not widely adopted by clinical audiologists (Vaerenberg et al., 2014; Browning et al., 2020; Sander et al., 2023). Clinical audiologists reportedly deactivate electrodes in the case of abnormal telemetry measures, evidence of extracochlear electrodes, or facial stimulation (Vaerenberg et al., 2014; Hemmingson and Messersmith, 2018), however, this practice is not carried over to measures of pitch ranking or electrode discrimination. A survey of CI practices in the United States indicated that 82% of audiologists agreed that deactivating electrodes based on pitch resolution could result in improved speech perception, but only 65% of respondents reported ever attempting electrode deactivation based on tonotopical tasks. Participants of this study indicated two primary barriers to implementing electrode deactivation strategies based on tonotopical tasks: (1) mixed evidence regarding benefit of electrode deactivation, and (2) a lack of a standardized methodology for identifying and deactivating indiscriminate electrodes (Sander et al., 2023). Similar practice patterns were reported in a global survey by Vaerenberg et al. (2014), clinical audiologists reported deactivating electrodes only about 10–15% of the time. Audiologists’ reasons for deactivating electrodes were largely tied to abnormal impedances and rarely based on behavioral feedback such as tonotopical tests (Vaerenberg et al., 2014). Thus, there is a need for more evidence to better understand the characteristics associated with improvements in outcomes following electrode deactivation, as well as evidence-based clinical strategies for creating and evaluating programs with optimized electrode configurations.

The purpose of this study was to evaluate the benefit of deactivating poorly encoding electrodes using a pitch ranking task. In this study, we exclusively used clinical tools available in the United States to identify and deactivate indiscriminate electrodes. Participants were tested in a baseline and experimental program immediately following the creation of the experimental program, and again following a 3–6 week acclimation period. It was hypothesized that the experimental program would result in significant benefit for adult CI users, measured by speech perception performance and user preference.

## 2. Materials and methods

### 2.1. Design

This study was conducted as a prospective within-subject repeated measures design. Participants were recruited to complete an electrode pitch ranking task to identify the presence of indiscriminate electrode pairs. Following the pitch ranking task, an experimental program was created where one or more electrodes related to indiscriminate pairs were selectively deactivated. Participants performed speech perception testing in their baseline condition (minimal changes made to their clinical program), with the experimental program prior to an acclimation period, and with the experimental program following a 3–6 week acclimation period. Participants also completed a preference-ranking task at each visit. Participants were blinded to the program conditions for all evaluations.

### 2.2. Participants

**FIGURE 1:**
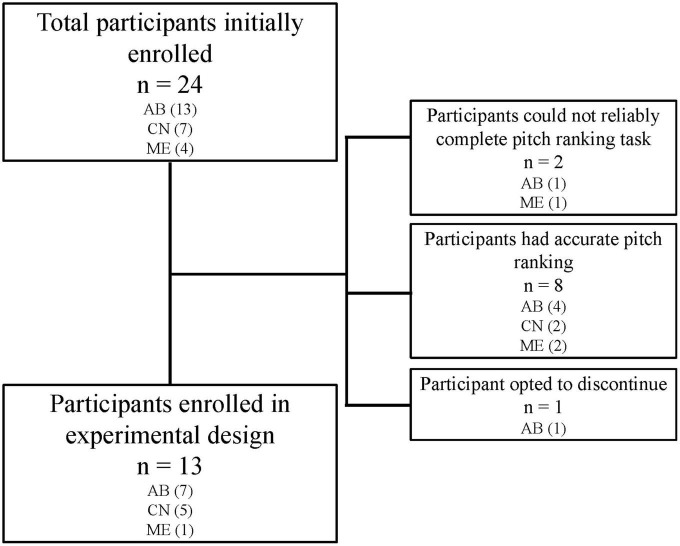
While 24 participants were initially enrolled in the study, only 13 participants completed the experimental study. Two participants could not reliably complete the pitch ranking task and therefore did not have electrodes deactivated. Eight participants reported pitch ranking which matched tonotopic organization of the cochlea and therefore had no indiscriminate pairs to consider for deactivation. One participant discontinued for reasons unrelated to the study protocol.

Twenty-four adult CI participants were initially recruited for this study. Two participants were excluded as they could not reliably report pitch-ranking, and 8 participants discontinued participation as they did not report any indiscriminate electrode pairs (i.e., they reported accurate pitch ranking). One participant reported indiscriminate pairs, but discontinued participation in the study due to reasons unrelated to the study protocol. Participants who completed the experimental design included thirteen adult CI users (11 male and 2 female), ranging in age from 20 to 81 years (mean = 54 years). Participant inclusion can be visualized in Figure 1. All participants were postlingually deafened adults who were native English speakers and had at least 1 year experience with their CI. Participants were capable of providing behavioral feedback and were deemed to have achieved stable hearing performance by their managing audiologists. Full-time use of the device (>8 h per day) was confirmed by datalogging. All participants screened negative for cognitive impairment by administration the Montreal Cognitive Assessment (MoCA) (Nasreddine et al., 2005). Devices from three commercial CI manufacturers were included in this study, including Advanced Bionics (AB; Valencia, CA, USA), Cochlear Nucleus (CN; Sydney, Australia), and MED-EL (ME; Innsbruck, Austria). Two participants were bilaterally implanted, and each ear was treated independently (S3/S10 and S11/S15). Each ear was evaluated in separate sessions, including unilateral speech perception testing and collection of sound perception questionnaire responses specific to the device. Participants had their contralateral ear plugged using a standard ear plug whenever contralateral acoustic hearing was present. Participant demographics can be found in Table 1.

**TABLE 1:** Patient demographics and electrode deactivations upon enrolling in the study.

No.	Sex	Age	Type	Electrode	Contra ear	Experience with CI	Etiology	No. of previously deactivated electrodes	Reason for previous electrode deactivation
S1	M	81	Advanced Bionics	HiFocus 1J	None	8 year 8 month	Acoustic neuroma	0	None
S3	M	81	Advanced Bionics	HiFocus 1J	CI	8 year 7 month	Unknown	1	Poor loudness growth (basal)
S8	M	80	Advanced Bionics	HiFocus 1J	CI	9 year 6 month	Genetic	3	Poor loudness growth (basal)
S9	M	54	MED-EL	Flex 28	None	3 year 3 month	Ototoxicity	0	None
S10	M	81	Advanced Bionics	HiFocus 1J	CI	7 year 10 month	Unknown	2	Poor loudness growth (basal)
S11	M	47	Cochlear Nucleus	Contour advance	CI	14 year 0 month	Unknown	1	Facial stimulation
S12	M	76	Cochlear Nucleus	Contour advance	CI	11 year 0 month	Noise exposure	0	None
S13	M	20	Cochlear Nucleus	Contour advance	None	16 year 11 month	Unknown	0	None
S15	M	47	Cochlear Nucleus	Contour advance	CI	8 year 0 month	Unknown	0	None
S17	M	27	Advanced Bionics	HiFocus 1J	HA	1 year 2 month	Unknown	0	None
S18	F	50	Advanced Bionics	HiFocus 1J	None	7 year 1 month	Unknown	0	None
S21	M	24	Advanced Bionics	HiFocus MS	HA	1 year 9 month	Unknown	0	None
S24	F	40	Cochlear Nucleus	Contour advance	HA	3 year 0 month	Ototoxicity	0	Poor loudness growth (basal)

Participants were compensated with a $25 gift card per appointment. This study was conducted in accordance with all applicable government regulations and University of Arkansas for Medical Sciences (UAMS) research policies and procedures. This protocol was approved by the UAMS Institutional Review Board (IRB) to conduct the study (IRB Protocol #205194).

### 2.3. Study intervention

Prior to pitch ranking measurements, the participants’ personal sound processor was connected to the most recent programming software available at the time of evaluation (AB Soundwave 2.3 and 3.0, CN Custom Sound 4.0, ME Maestro System Software 6.0). The device parameters of the sound processing algorithm (referred to as “program”) that the participant preferred to use in daily life (as set in their last clinical visit) served as an initial program for all programs used in this study. Any deactivated electrodes in the clinical program remained deactivated, and the reason for deactivation was recorded from clinical records (see Table 1). Telemetry confirmed no abnormal electrode impedances among active electrodes. Loudness balancing was performed at most comfortable levels (MCL) using a biphasic pulse train in the monopolar configuration to two adjacent electrodes sequentially with each electrode stimulating 2–5 pulses (Mathew et al., 2018; Biesheuvel et al., 2019). Current levels were adjusted to equal loudness across the electrode array, with the target loudness being “comfortably loud.” Adjustments were considered minimal from the participants’ clinical program (< 10 CUs for AB users, < 5 CL for Cochlear Nucleus users, and < 3% qu for MED-EL users). This loudness-balanced program based on the participants’ clinical program as the baseline condition (referred to as “baseline program”).

An experimental program was created from the participants’ baseline program. Testing of pitch ranking consisted of a two-alternative forced choice task comparing the participant’s perceived pitch of adjacent electrodes. The participant was asked to identify which electrode was higher in pitch. Stimulation was presented using pulse trains with an interstimulus interval of 0.5 s. Participants were trained using 2–4 trial runs comparing the most apical and basal active electrodes. Participants who did not demonstrate accurate pitch ranking in the training condition were dismissed at this time. Electrode pairs were compared in both the apical and basal direction, with a minimum of two stimulus sweeps in each direction. A pair was considered indiscriminate if the user reported the same pitch or a pitch-reversal on a majority of the comparisons (3 out of 4 confusions). In the case of an equal number of incorrect and correct responses (2 out of 4 incorrect responses), a tie-breaker comparison in each direction was presented. If there was still a tie, the electrode pair was not considered indiscriminate.

Once an electrode pair was determined to be indiscriminate, a decision was made regarding which electrode to deactivate. To make this decision, two programs were created wherein each electrode of the pair was deactivated. That is, for program A the apical electrode was deactivated, and for program B the basal electrode was deactivated. Each participant listened to a spoken passage using each program and was asked to select a preferred program. The preferred program was used as the experimental program. If the user had no preference, the most apical electrode was selected to be deactivated with the exception of the most basal two electrodes, in which the most basal electrode was deactivated. For participants who reported one or more indiscriminate electrode pairs, this process was systematically repeated with one pair investigated at a time, beginning with apical indiscriminate pairs, and moving basally. In cases where several indiscriminate electrodes were grouped together, a decision was made to leave the maximum number of electrodes activated.

After an electrode was deactivated, the program was presented to the user to confirm acceptability before considering additional indiscriminate electrode pairs, if any. Changes to the participants’ baseline program were kept to a minimum with the exception of the experimental change (i.e., electrode deactivation), which prompted the programming software to automatically reallocate the frequency bands of active electrodes and adjust the pulse width to keep the overall stimulation rate similar to that of the baseline program. The changes in pulse duration and rate were considered minimal. For CN users, the number of spectral maxima remained the same. For AB users, consideration was given to current-steering strategy utilized in their sound coding strategy. Each electrode had at least one adjacent electrode making it available to pair with another electrode. Slight adjustments in gain were applied for users who requested a volume comfort adjustment. This was typically implemented on electrodes adjacent to the deactivated electrode(s). The program which contained the deactivated electrodes is referred to as the “experimental program.” The experimental and baseline programs were compared, and slight adjustments were made to global loudness levels until both programs were reported to be perceived as equally loud.

### 2.4. Participant evaluation

Speech perception ability was measured using the participant’s baseline program and experimental program immediately following the establishment of the experimental program. Testing involved the administration of recorded materials including consonant-nucleus-consonant (CNC) test (Peterson and Lehiste, 1962) in quiet, and AzBio Sentences (Spahr et al., 2012) in quiet and in multitalker babble. All speech perception testing materials were presented according to parameters recommended by the Nilsson et al. (1996). Stimuli were presented at 60 dB SPL using a single loudspeaker at 0 degrees azimuth at a distance of 1 m in a double-walled, research-grade soundbooth. Calibration of the stimuli was performed daily at the input of the audiometer and the output of the loudspeaker. Signal-to-noise ratio (SNR) of the AzBio Sentences in noise was +5 dB SNR when applicable but was increased to +10 dB SNR when participants performed at floor effects (< 10% correct). Signal-to-noise ratio presentation levels were consistent within-user for all test conditions. Any contralateral devices were removed. Participants with any degree of contralateral residual hearing were fit with an ear plug for the duration of testing. Data from one participant (S24) was excluded from the speech in noise condition as their performance indicated a floor effect, bringing the analysis number to *n* = 12 for that condition.

To assess perceived benefit of the baseline and experimental program, participants were asked to rank subjective preference between the two programs using a visual 5-point Likert scale which ranged from “strongly prefer program A” to “strongly prefer program B,” with “no preference” being the center option. The preference assessment was administered within 1 h of the creation of the experimental program. For the preference assessment, the participants were blinded to the programs which were presented in random order. The user listened to a passage spoken by the tester, their own voice, and the voice of a companion if available. After several opportunities to hear each program, the participant was asked to fill out the comparison form.

Participants returned for follow-up testing after a 3–6 week acclimation period where the participant only had access to the experimental program. Data logging confirmed full-time use of the experimental program with each participant. Speech perception assessments were readministered using the same conditions described above. Participants again completed the blinded preference assessment. Following all testing, participants were also allowed to subjectively describe their experiences in freeform.

### 2.5. Statistical analysis

Statistically significant benefit in speech perception scores at the individual level was defined by critical difference values determined in Spahr et al. (2012) for AzBio Sentences and Carney and Schlauch (2007) for CNC words using a binomial distribution statistic for individual speech perception metrics. Group outcomes with the baseline program and experimental programs before and after the acclimation period were compared and analyzed using a non-parametric Friedman test of differences among repeated measures using SPSS Statistics software (IBM, Chicago, USA). Group change was deemed significant if it fell outside the 95th percentile confidence interval (*p* = 0.05). *Post hoc* analysis was determined using Wilcoxon sign-ranked testing with a Bonferroni correction applied. Participant preferences are reported descriptively.

Based on the number of CI users available to the researchers at the time of this study, it was estimated that the sample size would range between 20 and 30 participants. Twenty-four participants were initially recruited, but ten participants did not qualify for the experimental protocol and one participant opted to discontinue involvement. A total of 13 participants were included in group analysis. An analysis to determine effect size was conducted using G*Power version 3.1.9.7 (Faul et al., 2007) using the criteria of *n* = 13 participants, a significant criterion of α = 0.05, and a power = 0.8. These criteria yield an effect size of 0.85, which is a large effect size as interpreted by Cohen’s d (Cohen, 1992). For the group results related to speech in noise, the participant pool decreased to *n* = 12 participants. Using the same criteria, this yields an effect size of 0.89 (large effect size).

## 3. Results

### 3.1. Speech perception

**FIGURE 2:**
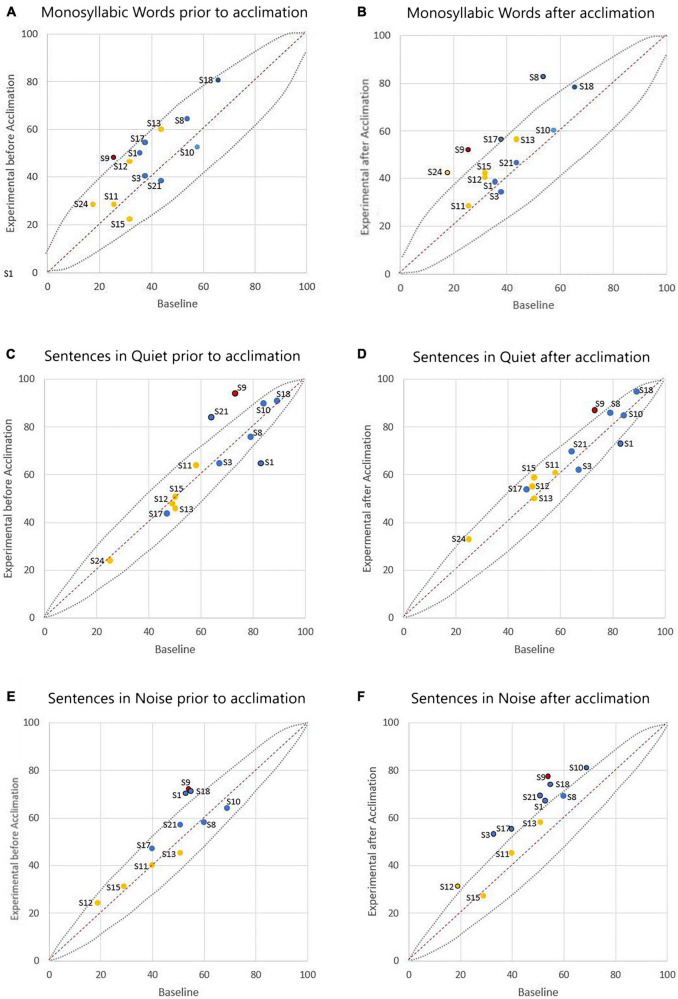
Comparison of individual speech perception scores (in percent correct) with the participant’s baseline program versus the experimental program. The left column **(A,C,E)** represents outcomes prior to the acclimation period, and the right column **(B,D,F)** represents outcomes following the acclimation period. The dashed lines show 95% confidence intervals for each test. Scores above the dashed line indicate statistically better performance with the experimental program. Scores below the dashed line indicate statistically poorer performance with the experimental program. Scores within the dashed lines indicate statistical equivalence. Data is colored to note cochlear implants (CI) device used (blue indicating AB, yellow indicating CN, and red indicating ME). Data point labels represent the participant number.

Figure 2 shows the individual speech perception results with the experimental program compared to the baseline program both before and following the acclimation period. Figures 2A, C, E display results with the baseline program and experimental program prior to the acclimation period. Four out of 13 participants had statistically better performance with the experimental program when compared to the baseline performance on at least one measure of speech perception. Specifically, one participant (S9) had a statistically significant improvement in CNC words. Two participants (S9, S21) had a statistically significant improvement in AzBio Sentences in quiet, and one participant performed significantly worse in the same condition (S1). Three participants (S1, S9, S18) had statistically significant improvement in AzBio Sentences in noise. All other participant performance was considered statistically equivalent.

Figures 2B, D, F display results with the baseline program and experimental program following the acclimation period. Ten out of 13 participants had statistically significant improvement on at least one measure of speech perception when compared to their performance with the baseline program. Four participants (S8, S9, S17, S24) had a statistically significant improvement in CNC words. One participant (S9) had a statistically significant improvement in AzBio Sentences in quiet, and again one participant performed significantly worse in the same condition (S1). Eight participants (S1, S3, S9, S10, S12, S17, S18, S21) had statistically significant improvement in AzBio Sentences in noise.

**FIGURE 3:**
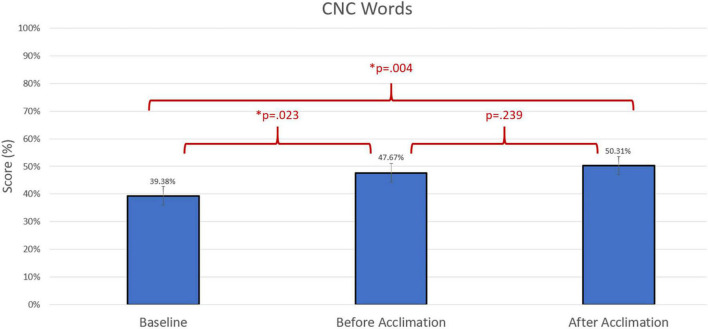
Comparison of average group performance on consonant-nucleus-consonant (CNC) words using the baseline and experimental program before and after the acclimation period.

**FIGURE 4:**
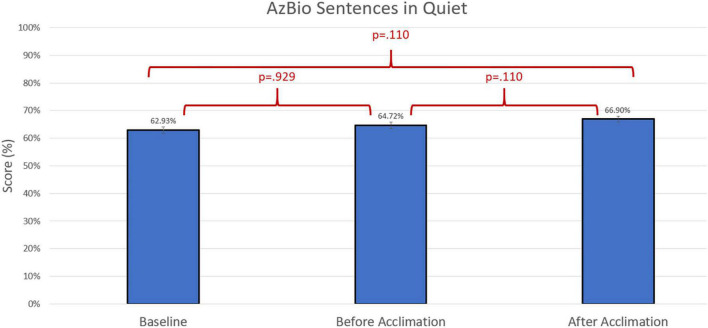
Comparison of average group performance on AzBio Sentences in quiet using the baseline and experimental program before and after the acclimation period.

**FIGURE 5:**
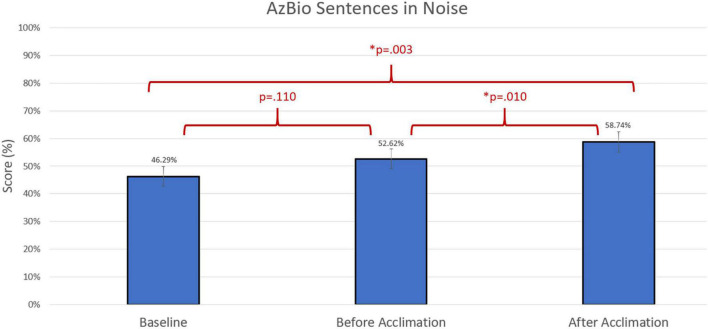
Comparison of average group performance on AzBio Sentences in noise using the baseline and experimental program before and after the acclimation period.

Group differences were compared using non-parametric repeated measures statistics due to small sample size (*n* = 13 for speech in quiet testing and *n* = 12 for speech in noise testing). Friedman’s test indicated group differences for CNC words (*p* = 0.006). A Bonferroni *post-hoc* test indicated an improvement in group performance with experimental program compared to baseline both prior to the acclimation period (*p* = 0.023) and following the acclimation period (*p* = 0.004). There were no group differences in group performance measured by AzBio Sentences in quiet (*p* > 0.05). Group differences were also indicated in the evaluation of AzBio Sentences in noise (*p* = 0.020). A Bonferroni *post-hoc* test indicated group improvements with the experimental program following the acclimation period compared to baseline (*p* = 0.003). Additionally, further improvement with the experimental program was found when comparing performance with the experimental program after the acclimation period compared to performance with the experimental program before the acclimation period (*p* = 0.010). Group differences can be found in Figures 3–5.

### 3.2. Patient preference

**FIGURE 6:**
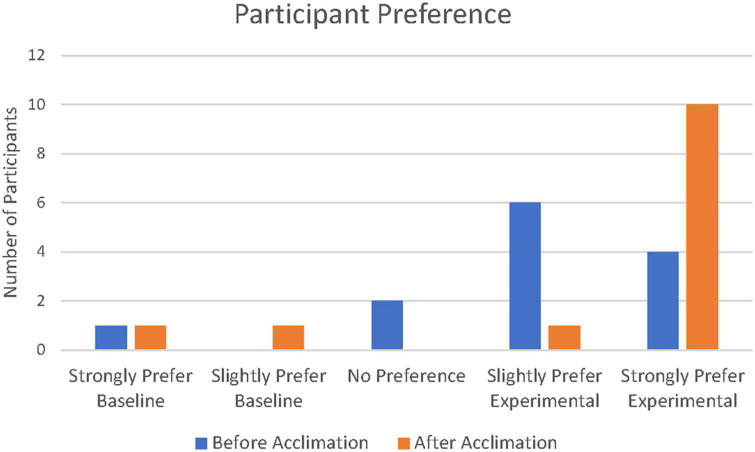
Participant preferences on a blinded preference evaluation comparing the baseline program to the experimental program. Blue bars represent preference prior to acclimation and orange bars represent preference after acclimation.

Prior to the acclimation period, four out of 13 participants strongly preferred the experimental program and six participants slightly preferred the experimental program. Two participants stated they had no preference and one participant stated they strongly preferred their baseline program. After the acclimation period, 10 out of 13 strongly preferred the experimental program, one participant slightly preferred the experimental program, one participant slightly preferred the baseline program, and one participant strongly preferred the baseline program. Patient preferences can be found in Figure 6. A summary of patient demographics, performance, and preference can be found in Table 2.

**TABLE 2:** A summary of participant results.

No.	Type	No. of indiscriminate pairs	Total no. active electrodes	CNC words		AzBio quiet		AzBio noise		Preference	
				pre-	post-	pre-	post-	pre-	post-	pre-	post-
S1	AB	3	13	o	o	−	−	+	+	+	−
S3	AB	1	15	o	o	o	o	x	+	−−	+ +
S8	AB	2	11	o	+	o	o	o	o	+	+ +
S10	AB	3	11	o	o	o	o	o	+	+	−−
S17	AB	4	12	o	+	o	o	o	+	+	+ +
S18	AB	1	14	o	o	o	o	+	+	+	+ +
S21	AB	2	14	o	o	+	o	o	+	o	+ +
S9	ME	1	11	+	+	+	+	+	+	o	+ +
S11	CN	4	17	o	o	o	o	o	o	+ +	+ +
S12	CN	2	20	o	o	o	o	o	+	+ +	+ +
S13	CN	3	19	o	o	o	o	o	o	+	+
S15	CN	4	18	o	o	o	o	o	o	+ +	+ +
S24	CN	2	20	o	+	o	o	x	x	+ +	+ +

Participants are grouped by device manufacturer. The terms “pre” and “post” indicate evaluation in relation to the acclimation period. The symbol “+” indicates significant improvement with the experimental program on that assessment. The symbol “−” indicates significantly worse scores with the experimental program on that assessment. The symbol “x” indicates this condition was not tested. The symbol “o” indicates that the user’s performance was statistically equivalent with both programs. For participant preference, “+ +” indicates strong preference for experimental, “+” indicates a slight preference for experimental, “o” indicates no preference, “−” indicates a slight preference for baseline, and “−−” indicates a strong preference for baseline.

## 4. Discussion

In this study, we investigated a clinical technique for pitch ranking to identify and deactivate poorly encoding electrodes in adult CI users. Findings from this study add to the literature describing methods of identification and selective deactivation of electrodes, as well as individual improvements following selective electrode deactivation and frequency reallocation. Among participants with indiscriminate electrode pairs, significant group improvements were noted after an acclimation period with monosyllabic words and sentences in noise, but not sentences in quiet. Additionally, when results are viewed at the individual level, speech perception results were statistically better with the experimental program on at least one measure in a majority of users following an acclimation period. In terms of user preferences, eleven out of 13 users preferred or strongly preferred the experimental program at the end of the study. These results are consistent with previous literature which found improvement in speech perception and sound quality using various methods of electrode identification and deactivation based on indications of poorly encoding electrodes (Zwolan et al., 1997; Saleh et al., 2013; Noble et al., 2014; Danieli et al., 2021). In this study, only one user had a decrease on any measures of speech perception, which is fewer than in the findings of Henshall and McKay (2001), Vickers et al. (2016), and Debruyne et al. (2017). Our findings are unique in that we implemented a strategy based in commercially available CI programming software using a technique that can be implemented directly into clinical practice.

### 4.1. Factors of influence

A difference in outcomes across manufacturers was anticipated due to the manufacturer-specific device design and signal coding strategies which are unique to each company. Channel independence related to electrode design is well documented (Berg et al., 2019, 2020, 2021) which provides context for the number of participants with indiscriminate pairs in each device group (see Figure 1). While three ME participants were initially recruited to participate in this study, only one participant had an indiscriminate electrode pair. This is likely attributed to the larger inter-electrode distance utilized in ME cochlear implant systems, allowing for fewer adverse effects of channel interaction when compared to devices which have a smaller electrode-to-modiolus distance (Berg et al., 2021), however, this observation should be regarded cautiously given the small number of ME participants evaluated in this study. AB and CN users unsurprisingly had a higher rate of indiscriminate pairs, given their electrode design which (1) is more proximal to the modiolus, and (2) contains more electrical contacts.

Table 2 provides a comprehensive view of outcomes grouped by device manufacturer. AB users had the highest percentage of users with improvement following electrode deactivation, with all seven users demonstrating significant benefit after the acclimation period on at least one measure. Nearly all AB users had an improvement in speech in noise perception following electrode deactivation. One AB user (S1) had a statistically worse performance on AzBio Sentences in quiet both before and after the acclimation period, but notably had a statistically significant improvement in AzBio Sentences in noise in the same program conditions. This was one of the two participants who preferred their baseline program after the acclimation period, suggesting that users are sensitive to a decrease in performance and may be able to indicate program preference related to performance. The second user who preferring the baseline program at the end of the study was also an AB user. All other AB users strongly preferred the experimental program at the end of the study.

Despite having largest number of indiscriminate electrode pairs (average of 3, range of 2–4), measurable improvement in speech perception was found less frequently with the experimental program among CN users. Only two out of the five CN device users demonstrated statistically significant improvement on any of the measures (S24 on CNC words, S12 on AzBio Sentences in noise). The findings with CN users are consistent with results of Henshall and McKay (2001), Vickers et al. (2016), and Debruyne et al. (2017) and is likely attributable to unique n-of-m stimulation strategy whereas only a select number of channels are activated in each cycle of stimulation. Four out of five CN users strongly preferred the experimental program at the end of the study, with one CN user slightly preferring the experimental program.

Only one participant in our study utilized a ME device, therefore the data should be considered with caution. Interestingly, this participant had only one electrode deactivated (most basal electrode) and demonstrated significant improvement on all measures of speech perception before and after acclimation. These device-specific findings may guide more research in manufacturer-specific guidelines for identifying and deactivating indiscriminate electrodes.

### 4.2. Participant preferences

Participants responded favorably to the experimental program. All participants found the experimental program to be tolerable through the acclimation period as evidenced by full time use of their device in the acclimation period. Most participants slightly preferred the experimental program prior to the acclimation period and shifted to a stronger preference following the acclimation period. Notably, the three participants who had no statistical improvement in speech perception preferred the experimental program both prior to and following the acclimation period, suggesting there may be benefits in sound quality in which speech perception testing is not sensitive. Research suggests that CI users have a bias toward a program in which they have the most experience (Tyler et al., 1986), therefore the general preference for the experimental program over the baseline program prior to acclimation is a surprising finding. The strong preference for the experimental program across most users supports the suggestion of benefit which was not adequately captured by speech perception testing. In addition to performance ratings, some participants provided unstructured feedback regarding the experimental program. Following the acclimation period, ten out of 13 participants provided unsolicited feedback that included the words “clear” or “clarity.” The informal reports on improvements in clarity supports the findings that spectral resolution is improved following electrode deactivation.

### 4.3. Clinical implications

Our study adds to the literature indicating that adult CI users have the potential to experience benefit by selectively deactivating electrodes associated with indiscriminate electrode pairs. Of clinical importance, 22 of the 24 recruited participants (91.7%) could reliably complete the pitch discrimination task which indicates good feasibility in adult populations. Of these 22 participants, 14 participants (63.7%) reported at least one indiscriminate electrode pair. This indicates that a moderately high rate of cochlear implant users with indiscriminate electrodes which can be identified using a clinical task. Despite the moderately high prevalence of poorly encoding electrodes and potential for individual improvement following deactivation, clinical audiologists do not commonly practice electrode deactivation (Vaerenberg et al., 2014; Browning et al., 2020; Sander et al., 2023). This is likely due to audiologists’ lack of access to a clinically feasible, evidence-based approach to electrode identification and deactivation (Sander et al., 2023). This clinical electrode deactivation task was found to fit easily in the clinical domain. Our pitch ranking task took between 12 and 15 min to perform, depending on manufacturer (CN requiring the most time due to having the most electrode comparisons, and ME requiring the least time due to having the least electrode comparisons). The user’s ability to remain attentive and confident in reporting channel discrimination also contributed to the amount of time needed to perform the pitch ranking task. All tools and measures are clinically available and only require the time committed to the pitch ranking task. Further work is needed to better understand how to integrate electrode deactivation into clinical practice. Questions to address may include how to identify the optimal number of electrodes needed for maximum CI benefit and when in the CI user’s treatment timeline to perform this task. Mathew et al. (2018) found that CI users demonstrated an improvement in electrode discrimination up to 12 months after initial activation, suggesting that electrode deactivation based on pitch ranking may not be appropriate until a CI user’s performance has stabilized.

Our study found speech perception performance and participant preference to increase over time with the experimental program. The number of individuals with statistically significant improvements in speech perception increased from 4 to 10 (out of 13) following the acclimation period of 3–6 weeks. This should signal to clinicians that validation of their changes may not be immediately apparent and require follow-up evaluation at a subsequent visit to confirm benefit.

Finally, this research has implications for future development of programming practices among audiologists who program cochlear implants. Participants in our study did not have corresponding post-operative imaging to compare to perceptual reports of pitch, however, the relationship between pitch discrimination and known electrode location as verified through CT scan is an interesting area for exploration. Additionally, this task could be considered in the application of programming auditory brainstem implants, as the arrangement of the channel-to-electrode relationship is challenging due to the less distinct tonotopic organization of the cochlear nucleus as compared to the cochlea.

### 4.4. Strengths and limitations

A strength of this study is it is the first of its kind to use clinically available equipment to deactivate electrodes based on a pitch ranking task, thus demonstrating the clinical feasibility of implementing this practice. An additional strength is that we used a prospective within-subject study design, with a follow-up after a 3–6 week acclimation period where the CI user exclusively utilized the experimental program.

Limitations of this study include a small sample size, which is consistent with CI research. We also did not have a control condition where participants had electrodes deactivated randomly, therefore we cannot entirely rule out the improvement being simply related to fewer channels instead of tonotopic-specific deactivations. Additionally, participants were not retested in their baseline condition after the acclimation period, which would have strengthened our results. In our participant population, there was a lack of equal distribution among CI device manufacturers which could not be balanced due to our available participant population. Additionally, while every effort was made to blind participants to testing conditions, participants were often aware of the conditions due to their familiarity with their baseline program. Blindness to program condition during the acclimation period could not be guaranteed for the same reason. While we acknowledge the bias of potential awareness of the new program, prior research suggests a bias for the program which the user has the most experience with (Tyler et al., 1986), thus, we would expect the experimental program to be at a disadvantage when compared to the baseline program.

## 5. Conclusion

The present study provides additional evidence to support electrode deactivation based on discrimination in an effort to improve speech perception. A majority of CI users had improved speech perception performance with the experimental program, and only one participant performed significantly worse on one measure. Additionally, a majority of CI users preferred the experimental program. This study supports the practice of pitch ranking and deactivation approaches in an effort to improve patient performance and satisfaction.

## Data availability statement

The raw data supporting the conclusions of this article will be made available by the authors, without undue reservation.

## Ethics statement

The studies involving human participants were reviewed and approved by the UAMS IRB. The patients/participants provided their written informed consent to participate in this study.

## Author contributions

SW was involved in protocol design, execution, coordination, and analysis of the study. SA was involved in protocol design of the study. Both authors contributed to manuscript revision, read, and approved the submitted version.
